# Preparation of Enantiomeric β-(2′,5′-Dimethylphenyl)Bromolactones, Their Antiproliferative Activity and Effect on Biological Membranes

**DOI:** 10.3390/molecules23113035

**Published:** 2018-11-20

**Authors:** Witold Gładkowski, Aleksandra Włoch, Aleksandra Pawlak, Angelika Sysak, Agata Białońska, Marcelina Mazur, Paweł Mituła, Gabriela Maciejewska, Bożena Obmińska-Mrukowicz, Halina Kleszczyńska

**Affiliations:** 1 Department of Chemistry, Wrocław University of Environmental and Life Sciences, Norwida 25, 50-375 Wrocław, Poland; marcelina.mazur@upwr.edu.pl; 2 Department of Physics and Biophysics, Wrocław University of Environmental and Life Sciences, Norwida 25, 50-375 Wrocław, Poland; aleksandra.wloch@upwr.edu.pl (A.W.); halina.kleszczynska@upwr.edu.pl (H.K.); 3 Department of Pharmacology and Toxicology, Wrocław University of Environmental and Life Sciences, Norwida 31, 50-375 Wrocław, Poland; aleksandra.pawlak@upwr.edu.pl (A.P.); angelika.sysak@upwr.edu.pl (A.S.); bozena.obminska-mrukowicz@upwr.edu.pl (B.O.-M.); 4 Department of Crystallography, University of Wrocław, Joliot Curie 14, 50-383 Wrocław, Poland; agata.bialonska@uwr.edu.pl; 5 Institute of Environmental Engineering, Wroclaw University of Environmental and Life Sciences, Grunwaldzki Sq 24, 50-363 Wrocław, Poland; pawel.mitula@upwr.edu.pl; 6 Central Laboratory of the Instrumental Analysis, Wroclaw University of Technology, Wybrzeże Wyspiańskiego 27, 50-370 Wroclaw, Poland; gabriela.maciejewska@pwr.edu.pl

**Keywords:** lactones, bromolactonization, absolute configurations, antiproliferative activity, hemolytic activity, erythrocytes, biological membranes

## Abstract

Three novel enantiomeric pairs of bromolactones possesing a 2,5-dimethylphenyl substituent at the β-position of the lactone ring have been synthesized from corresponding enantiomeric (*E*)-3-(2′,5′-dimethylphenyl)hex-4-enoic acids (**4**) by kinetically controlled bromolactonization with *N*-bromosuccinimide (NBS). γ-Bromo-δ-lactones (**5**) were isolated as the major products. Absolute configurations of stereogenic centers of γ-bromo-δ-lactones (**5**) were assigned based on X-ray analysis; configurations of *cis* δ-bromo-γ-lactones (**6**) and *trans* δ-bromo-γ-lactones (**7**) were determined based on mechanism of bromolactonization. Synthesized compounds exhibited significant antiproliferative activity towards the four canine cancer cell lines (D17, CLBL-1, CLB70, and GL-1) and one human cancer line (Jurkat). Classifying the compounds in terms of activity, the most active were enantiomers of *trans* δ-bromo-γ-lactones (**7**) followed by enantiomers of *cis* isomer (**6**) and enantiomeric γ-bromo-δ-lactones (**5**). Higher activity was observed for all stereoisomers with *S* configuration at C-4 in comparison with their enantiomers with 4*R* configuration. Synthesized compounds did not induce hemolysis of erythrocytes. The results of the interaction of bromolactones with red blood cell membranes suggest that these compounds incorporate into biological membranes, concentrating mainly in the hydrophilic part of the bilayer but have practically no influence on fluidity in the hydrophobic region. The differences in interactions with the membrane between particular enantiomers were observed only for γ-lactones: stronger interactions were found for enantiomer 4*R*,5*R*,6*S* of *cis* γ-lactone (**6**) and for enantiomer 4*S*,5*R*,6*S* of *trans* γ-lactone (**7**).

## 1. Introduction

Cytotoxic activity against different cancer cell lines is one of the most characteristic biological activities of lactones possessing an aromatic ring in their structures. Among this diverse group of compounds many reports concern antiproliferative activity of natural lactones i.a. coumarin [1], campthotecin [2], (−)-cleistenolide [3], (+)-crassalactones [4,5], or styryl lactones [6], including goniothalamin [7] or 5,6-dehydrokavain [8]. Lignan lactones, known for their potent antiproliferative activity, are secondary metabolites isolated from dietary or medicinal plants containing naphtalene unit [9] or dibenzylbutyrolactone framework i.a. isohaichulactone [10], matairesinol [11], 7-hydroxymatairesinol [12], mammalian lignan enterolactone [13]. Numerous structural analogs of these bioactive molecules, in some cases exhibiting comparable or higher activity than their parent compounds, have also been designed and synthesized, such as novel derivatives of coumarins: dihydropyrazole thio-ethanone derivatives [14], α-methylidene-δ-lactones with 3,4-dihydrocoumarin skeleton [15], series of 4-substituted coumarins [16] or coumarin/2-cyanoacryloyl hybrids [17]. Various antitumor analogs of isohaichulactone [18], homocampthotecin [19] and styryl lactones i.a. (+)-crassalactones B and C [20] or goniofufurone [21] have been also prepared.

Compounds bearing a β-aryl-γ-lactone or β-aryl-δ-lactone core derived from aromatic aldehydes also exhibited cytotoxic effects on different cancer lines. Significant activity against human promyelocytic leukemia (HL-60) line was shown for racemic β-aryl-δ-iodo-γ-lactones obtained in five-steps synthesis in which the first step was Doebner condensation of aromatic aldehydes with monomethyl malonate [22]. Exploring the effect of chirality on the activity of tested compounds, we have prepared recently new series of racemic and enantiomerically enriched β-aryl-δ-iodo-γ-lactones with defined configurations of stereogenic centers. Synthesized lactones showed antineoplastic activity against canine cancer lines, namely D17 (canine osteosarcoma), GL-1 (B cell leukemia) and CLBL-1 (B cell lymphoma) [23,24] Furthermore, both enantiomers of *trans*-δ-iodo-γ-lactones possessing 2,5-dimethylphenyl ring were proved to induce caspase-dependent apoptosis through downregulation of the expression of antiapoptotic proteins Bcl-xL and Bcl-2 [25].

Searching for the active compounds as potential drug candidates in the chemotherapy of cancer, and encouraged by the results obtained for optically active iodolactones derived from 2,5-dimethylbenzaldehyde, in this work we report a synthesis of their chiral bromo analogs and evaluation of their activity against panel of canine cancer lines.

Chemotherapeutic agents may act through various mechanisms leading to apoptosis. Because their main targets are intracellular substructures in the first step they must penetrate the outer cell membrane [26]. Thus, understanding the interactions between anticancer drugs and cell membranes is an important area of research because of its direct relationship with pharmacological activity of the compounds. For that reason, we expand the goals of this work by the studies on interactions of synthesized bromolactones with cell membranes. For the preliminary studies reported herein, we chose red blood cell membranes as good models of such investigations.

## 2. Results and Discussion

### 2.1. Synthesis of Enantiomeric Bromolactones (***5***–***7***)

**Scheme 1:**
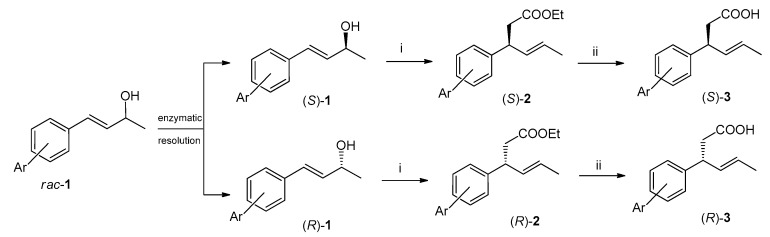
Synthesis of enantiomeric (*E*)-3-(2′,5′-dimethylphenyl)hex-4-enoic acids [(*S*)-**3** and (*R*)-**3**]. Reactions and conditions: (i) CH_3_C(OEt)_3_, C_2_H_5_COOH, 138 °C and (ii) NaOH, EtOH, reflux.

In our previous research on antiproliferative compounds the convenient synthesis of enantiomerically pure or enriched iodolactones containing β-aryl substituents was elaborated. The key step of the synthetic pathway was kinetic resolution of racemic (*E*)-4-arylbut-3-en-2-ols (**1**) via transesterification catalyzed by lipase B from *Candida antarctica*, followed by Johnson–Claisen rearrangement of enantiomeric alcohols to γ,δ-unsaturated esters (**2**) [24,27,28]. Their hydrolysis afforded the corresponding (*S*)- and (*R*)-acids (**3**) (Scheme 1) which were the direct substrates of iodolactonization. 

**Scheme 2:**
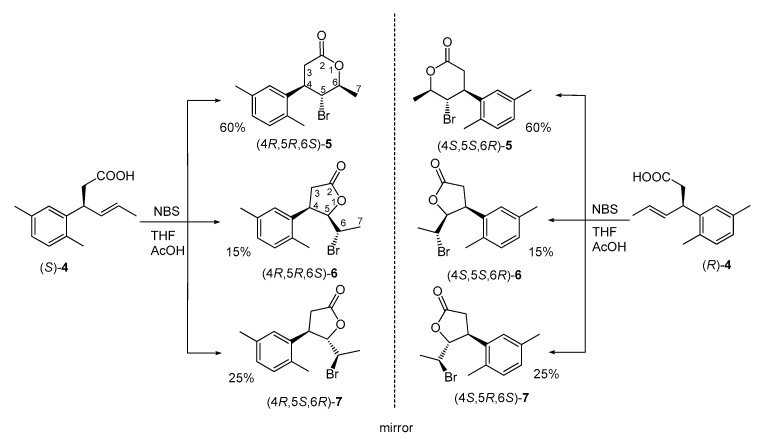
Bromolactonization of enantiomeric (*E*)-3-(2′,5′-dimethylphenyl)hex-4-enoic acids [(*S*)-**4** and (*R*)-**4**] (% of bromolactones according to GC)

In the studies described herein, enantiomerically pure (*S*)- and (*R*)-3-(2′,5′-dimethylphenyl)hex-4-enoic acids (**4**) were used independently as substrates for bromolactonization with NBS to obtain enantiomeric pairs of bromolactones (Scheme 2) as the analogs of previously reported iodolactones possessing 2,5-dimethylphenyl ring [24]. In both reactions three products (**5**–**7**) were detected, separated, and purified by silica gel column chromatography. Their structures were established based on spectroscopic methods, including IR and NMR spectroscopy.

**Figure 1:**
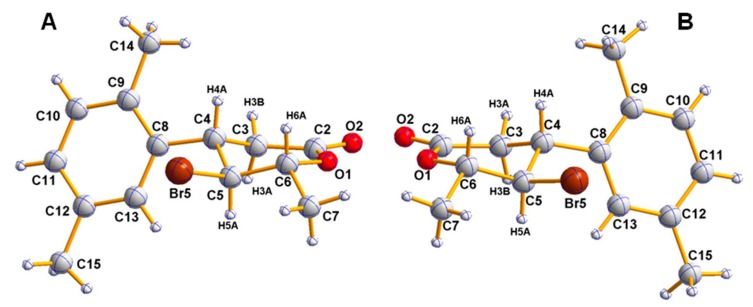
Crystal structures of two enantiomers of γ-bromo-δ-lactones **5**: 4*S*,5*S*,6*R* (**A**) and 4*R*,5*R*,6*S* (**B**) with crystallographic numbering.

The major products of bromolactonization (60% in the reaction mixture according to GC) were the most polar ones (R_f_ = 0.33). Analysis of their IR spectra showed strong C=O absorption bands at 1720 cm^−1^ confirming the presence of δ-lactone ring. Excluding signals within the range of 6.92 to 7.08 ppm coming from protons at aromatic ring, the most shifted downfield on ^1^H NMR spectrum were doublet of quartets from H-6 proton at 4.68 ppm as well as triplet at 4.05 ppm from H-5. Their locations on NMR spectra were the result of deshielding effect of the oxygen and bromine atom, respectively. Furthermore, the coupling constant of proton H-5 with proton H-4 and H-6 (*J* = 10.8 Hz) suggested the pseudoaxial orientation of these three protons and hence the pseudoequatorial orientations of substituents at C-4, C-5, and C-6 in half-chair like conformation of six-membered ring. Conclusions drawn from the spectroscopic data were confirmed by X-ray analysis. Obtained crystal structures present enantiomeric γ-bromo-δ-lactones (**5**) with the bromine at C-5 located *trans* towards both benzene ring at C-4 and methyl group at C-6 (Figure 1).

Two minor products with lower polarity isolated after bromolactonization of enantiomeric acids (**4**) were identified as *cis*-δ-bromo-γ-lactones (**6**) (R_f_ = 0.47) and *trans*-δ-bromo-γ-lactones (**7**) (R_f_ = 0.38). They made up 15% and 25% of the reaction mixture, respectively. Spectroscopic data of these compounds were very similar to those of their iodo analogs reported previously [24]. 

In the case of bromolactonization described herein, heterolytic cleavage of NBS to bromide cation (Br^+^) and succinimide anion is followed by the addition of Br^+^ to the double bond of acid **4** and convertion of the carboxyl group into carboxylate by succinimide anion [29,30]. Contrary to the previously reported iodolactonization of enantiomeric acids **4** in which major products were γ-lactones [24], during bromolactonization we observed preferential formation of the product of 6-*exo* cyclization, γ-bromo-δ-lactone **5** over the products of 5-*endo* cyclization, δ-bromo-γ-lactones (**6**) and **7**. The dependence of halolactonization regioselectivity on the electrophile species was observed by us earlier for series of 3-arylhex-4-enoic acids [23]. Previous investigations of Snider and Johnston [31] showed that the nature of the electrophile and the substrate structure is decisive for regioselectivity in halolactonizations. In their studies bromolactonization of the series of γ,δ-unsaturated acids also afforded significantly more δ-lactones than iodolactonization. This difference was explained based on the observations made by the group of Williams et al. [32]. They proved that in bromocyclization a rate determining step is the addition of bromine to the double bond, while in iodocyclization limiting step is the attack of the nucleophile on the iodine-double bond complex.

**Scheme 3:**
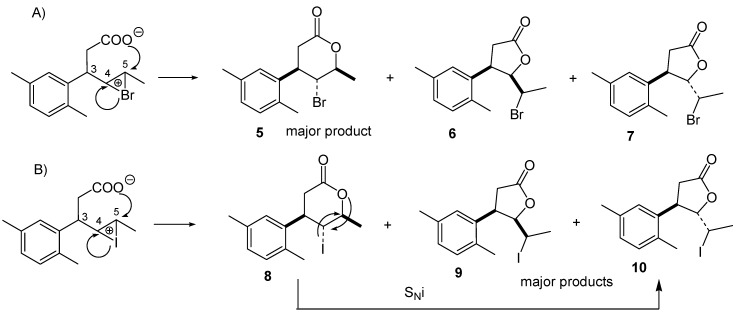
Bromolactonization (NBS, THF) under kinetic control (**A**) and iodolactonization (I_2_, NaHCO_3_/Et_2_O) under thermodynamic control (**B**) leading to different major products from acid **4.**

Taking into consideration these findings, bromolactonization with NBS described herein is under kinetic control (Scheme 3A). Steric and electronic repulsions between aryl substituent at C-3 and carboxylate ion hinder attack on C-4 thus favoring formation of six-membered ring. As a result, δ-lactone forms faster than γ-lactone and the former predominates in the product mixture. In the case of thermodynamically controlled iodolactonization (I_2_, NaHCO_3_, Et_2_O), the rapidly-formed δ-lactone is easily rearranged to the more thermodynamically stable γ-lactone via 1,2-migration of iodine with simultaneous formation of 5-membered ring (Scheme 3B). Similar mechanism of double internal nucleophilic substitution (S_N_i) in which the oxygen of the lactone ring attacks the halogen-bounded carbon from the opposite site of iodine was reported by Holbert et al. [33] during stereospecific rearrangement of monocyclic iodo-β-lactones to more thermodynamically stable γ-lactones. Analysis of this rearrangement for γ-iodo-δ-lactone (**8**), carried out on Dreiding model, indicated that such steric course of this isomerization leads to isomer *trans* of δ-iodo-γ-lactone (**10**).

**Scheme 4:**
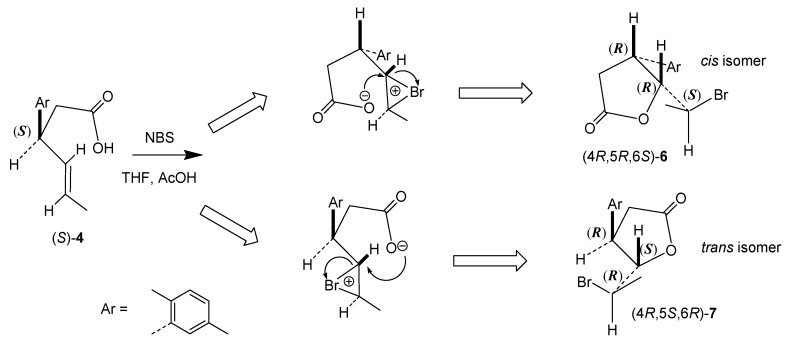
Mechanism of bromolactonization of (*S*,*E*)-3-(2′,5′-dimethylphenyl)hex-4-enoic acid (**4**) determining the absolute configurations of stereogenic centers in γ-lactones **6** and **7**.

Configurations of stereogenic centers for both enantiomers of *cis* bromolactones **6** and *trans* bromolactones **7** synthesized herein were assigned taking into account the steric course of bromolactonization. Configuration *R* at C-4 in both stereoisomers after lactone ring closure is determined by the absolute configuration of starting acid (*S*)-**4**. Configurations of newly formed chiral centers at C-5 and C-6 are the consequence of nucleophilic attack of the carboxylate ion at C-5 from the opposite side of the bromonium ion, leading to antiperiplanar orientation of the C-O and C-Br bonds. As a result, configurations 5*R* and 6*S* for *cis* isomer as well as 5*S* and 6*R* for *trans* isomer are ascribed (Scheme 4). Likewise, in the case of γ-lactones formed from (*R*)-acid **4**, isomer *cis* possesses configurations 4*S*,5*S*,6*R* and isomer *trans*-4*S*,5*R*,6*S*. 

**Scheme 5:**
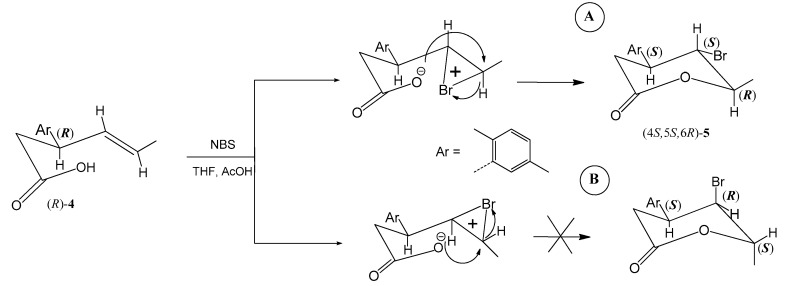
Mechanism of bromolactonization of (*R*,*E*)-3-(2′,5′-dimethylphenyl)hex-4-enoic acid (**4)** explaining formation of stereoisomer 4*S*,5*S*,6*R* of δ-lactone **5**.

Absolute configurations of all stereogenic centers in both synthesized enantiomeric γ-bromo-δ-lactones (**5**) (Figure 1) were assigned by the structure determination of these compounds containing a chiral reference molecule of known absolute configuration at C-4 and confirmed by anomalous dispersion effects in diffraction measurements on the crystals. As a result, configurations 4*S*,5*S*,6*R* were ascribed to the enantiomer of δ-lactone **5** obtained from (*R*)-acid **4** and opposite configurations to the enantiomer produced from (*S*)-acid **4**. These assignments were fully consistent with the absolute configurations predicted on the basis of the bromolactonization mechanism (Scheme 5, mechanism shown only for (*R*)-acid **4**) as well as with absolute configurations of their enantiomeric iodo analogs with phenyl and 4-methylphenyl ring, determined by X-ray analysis [27]. 

Formation of only one stereoisomer of γ-bromo-δ-lactone **5** (A) during antiperiplanar opening of halonium ion is favored during 6-*endo* cyclization of 3-arylhex-4-enoic acids [23]. Analysis of structure of second theoretical stereoisomer (B), in which the energetically unfavorable pseudoaxial positions at C-5 and C-6 would have to be occupied by bromine and methyl substituent respectively, explains its discrimination in the reaction course (Scheme 5).

### 2.2. Antiproliferative Activity

Cytotoxicity of enantiomeric pairs of bromolactones **5**–**7** against selected cancer cell lines was measured in vitro using 3-(4,5-dimethylthiazol-2-yl)-2,5-diphenyl tetrazolium bromide (MTT) assay. Four of the cell lines represent human (Jurkat) and canine (CLBL-1, CLB70 and GL-1) hematopoietic cancers, D17 is a canine osteosarcoma cell line. The results of the tests are shown in Table 1 as IC_50_ values.

All tested bromolactones (entries 1–6) exhibited noticeable concentration-dependent activity against examined hematopoietic cancer cell lines whereas only isomer *trans*-(4*S,*5*R,*6*S*)-**7** was active against the most resistant D17 cell line (entry 6). In half of 30 tests, IC_50_ values were in the range 6 to 23 µg/mL. In the case of human T cell leukemia (Jurkat) for most of γ-lactones **6,7** (entries 4–6) the activity was higher than the activity of carboplatin used as a control. In the case of canine B cell leukemia (GL-1), only isomers of γ-lactones with 4*S* configuration IC_50_ values were comparable with those determined for carboplatin (entries 4 and 6). Classifying the tested compounds in terms of decreasing activity, the most active were enantiomers of *trans*-5-(1-bromoethyl)-4-(2′,5′-dimethylphenyl)dihydrofuran-2-one (**7**) (entries 5,6) followed by enantiomers of *cis* isomer **6** (entries 3,4) and the less activity was found for enantiomeric γ-bromo-δ-lactones **5** (entries 1,2). The highest activity (IC_50_ < 10 µg/mL) was observed for lactone *trans*-(4*S*,5*R*,6*S*)-**7** against GL-1 and Jurkat as well as for lactone *cis*-(4*S*,5*S*,6*R*)-**6** against GL-1. In the case of B cell lymphoma (CLBL-1) and B cell chronic leukemia (CLB70) the most active were also stereoisomers (4*S*,5*R*,6*S*)-**7** and (4*S,*5*S,*6*R*)-**6**, respectively. Considering the effect of configuration of stereogenic centers one can see that in all cases enantiomers obtained from (*R*)-acid **4** [(4*S*,5*S*,6*R*)-**5**, (4*S*,5*S*,6*R*)-**6**, and (4*S*,5*R*,6*S*)-**7**)] were more active than their antipodes derived from (*S*)-acid **4**. This correlation is particularly observed for the activity of enantiomeric *trans* δ-bromo-γ-lactones **7** towards D17 cell line, in this case enantiomer 4*S*,5*R*,6*S* (entry 6) inhibited the proliferation whereas 4*R*,5*S*,6*R* was inactive (entry 5). For all tested enantiomeric pairs of bromolactones significant correlation between configurations of their stereogenic centers and cytotoxic effect was also found for GL-1 cell line; in this case the differences in activity between enantiomers were around 50%.

Comparing the antiproliferative potency of *cis* (**6**) and *trans* δ-bromo-γ-lactones (**7**) with their iodo analogs **9**,**10** reported earlier [24] (entries 7–10) the considerably higher activity of the latter towards CLBL-1 cell line is observed and contrary to bromolactones tested herein, all stereoisomers of δ-iodo-γ-lactones were active against D17 cell line. In the case of two remaining cancer cell lines, the activity of δ-bromo-γ-lactones **6**–**7** turned out to be generally higher than corresponding iodolactones. The differences between the enantiomers of bromolactones were significantly higher in comparison with iodolactones. Detailed studies on the antiproliferative effect of enantiomeric *trans* δ-iodo-γ-lactones with 2,5-dimethylphenyl ring at β position proved a significantly stronger induction of cell death for enantiomer 4*R*,5*S*,6*R* [25] whereas IC_50_ values determined in this work for *trans* δ-bromo-γ-lactones **7** indicated higher potency of enantiomer 4*S,*5*R,*6*S*. The same enantiomer of *trans* δ-iodo-γ-lactone with *p*-isopropylphenyl substituent at β-position was reported as more active against selected canine cancer cell lines in our previous work [34].

### 2.3. Hemolytic Activity

The hemolytic activity test was performed to determine the cytotoxicity of the tested compounds using pig red blood cells (RBCs). Results indicated that the tested bromolactones did not induce hemolysis in a range of concentrations from 10 to 100 µM (data not shown). The percentage of hemolysis was at control level and did not exceed 3%. The degree of in vitro toxicity of compounds is evaluated by testing hemolytic activity on the basis of observed mortality rate. The compound is classified as nontoxic if the hemolysis is in the range 0 to 9%, slightly toxic in the range 10 to 49%, and toxic in the range 50 to 89%. Hemolysis between 90 and 100% means a highly toxic compound [35]. Our results indicate that the compounds in the range of used concentrations do not have a toxic effect on red blood cells.

### 2.4. Fluorescence Spectroscopy

**Figure 2:**
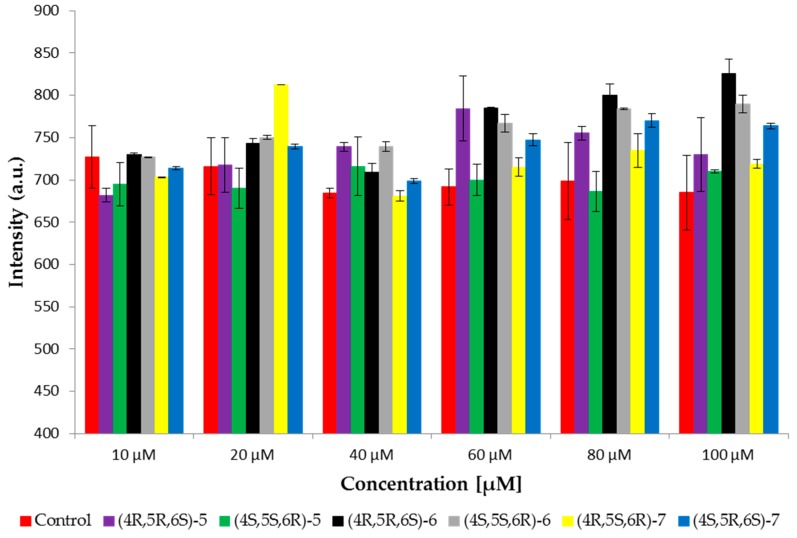
Changes of intensity of MC540 probe measured at 585 nm at 37 °C for RBCM (control) and RBCM with addition of compounds.

Fluorescence spectroscopy was used to study the effect of tested compounds on red blood cell membranes (RBCM). Three fluorescent probes were used: MC540, Laurdan, and DPH. MC540 provides information about the interface of membrane, because the negative charge of the probe is located slightly above the domain of the glycerol backbone [36,37]. Changes in fluorescence emission of the probe provide information about lipid spacing. When the phospholipids are closely spaced, the maximum of emission intensity occurs at about 621 nm. MC540 has a maximum intensity at approximately 585 nm, where lipids are more loosely packed and the intermolecular spacing is therefore greater [38,39]. In our studies the fluorescence emission from probes in the range 560 to 660 nm was examined. The results showed the maximum emission at 585 nm. Thus the maximum has been read for each sample at 585 nm and compared to the control samples. Figure 2 shows the maximum intensity of MC540 probe at six concentrations of tested bromolactones. Results showed increased intensity with increasing concentration of the enantiomer 4*R*,5*R*,6*S* of δ-lactone **5**; both enantiomers of *cis* γ-lactone **6** and enantiomer 4*S*,5*R*,6*S* of *trans* γ-lactone **7**. The highest increase of intensity was observed for enantiomer 4*R*,5*R*,6*S* of *cis* γ-lactone **6** whereas the intensity of enantiomer 4*S*,5*S*,6*R* of *cis* γ-lactone **6** and enantiomer 4*R*,5*S*,6*R* of *trans* γ-lactone **7** was at control level. Studies have shown that differences in fluorescence intensity result from differences in probe adsorption to the lipid bilayer with different degree of order. The increase in fluorescence intensity of MC540 probably indicates a change in lipid packing, which would mean a greater membrane surface area available for the binding of the dye [40]. 

The tested compounds, in particular enantiomer 4*R*,5*R*,6*S* of *cis* γ-lactone **6**, change the packing of lipids on the surface on the membrane, that indicates interaction with this part of the membrane.

Another fluorescent probe used in the study was Laurdan which provides information about hydrophobic–hydrophilic regions of the phospholipid bilayers (at the level of the phospholipid glycerol backbone). This probe is also sensitive to polarity changes and dynamic properties at the membrane lipid-water interface [41,42]. Changes in the polar group packing arrangement of the membrane were investigated on the basis of generalized polarization (GP) of the Laurdan probe. 

**Figure 3:**
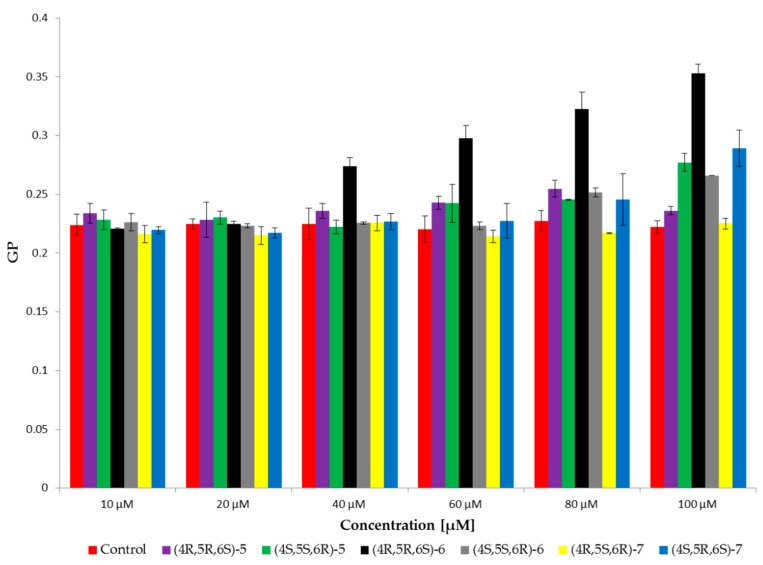
Values of generalized polarization (GP) of Laurdan probe for the RBCM (control) and RBCM with addition of compounds at 37 °C.

The results showed an increase in values of GP for enantiomer 4*R*,5*R*,6*S* of *cis* γ-lactone **6** from concentration 40 µM. On the other hand, the value of GP for enantiomer 4*R*,5*S*,6*R* of *trans* γ-lactone **7** was at the control level. For the rest of tested bromolactones values of GP increased from concentration 60 µM (Figure 3). It is evident that in the presence of enantiomer 4*R*,5*R*,6*S* of *cis* γ-lactone **6** changes in GP are more pronounced than for other compounds accounting for approximately 60% of the control. An increase in the value of GP probably indicates a decrease in water content in the area of glycerol with a corresponding increase in the order in hydrophobic–hydrophilic regions of the phospholipid bilayers. 

Changes in fluidity of the hydrophobic regions of the phospholipid bilayers were determined on the basis of changes in fluorescence anisotropy (A) of DPH probe. The increased anisotropy indicates increased order in the hydrophobic area (within the hydrocarbon chains) and the decrease in the value of anisotropy testifies about increased fluidity of the membrane.

**Figure 4:**
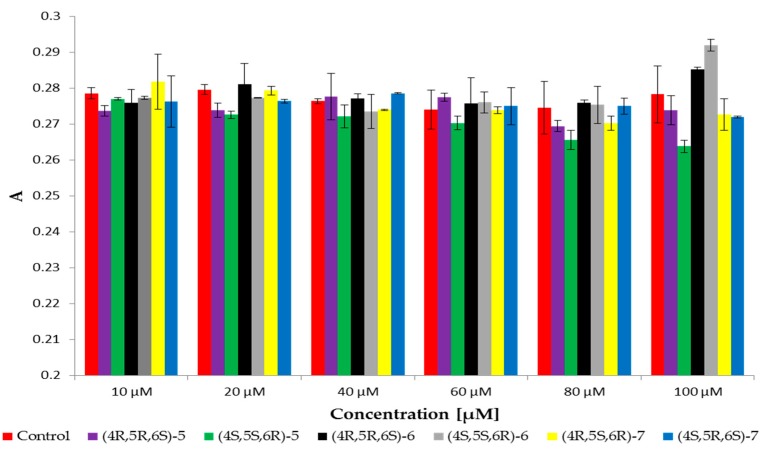
Values of anisotropy (A) of DPH probe for RBCM (control) and RBCM with addition of compounds at 37 °C.

The results indicated that enantiomer 4*S*,5*S*,6*R* of *cis* γ-lactone **6** induced slightly increased anisotropy at highest concentration. It caused a slight stiffening of the membrane in that area. For its antipode-(4*R*,5*R*,6*S*)-**6** and both enantiomers of *trans* γ-lactone **7** the values of anisotropy were at the level of control (Figure 4). It is interesting that enantiomer 4*R*,5*R*,6*S* of δ-lactone **5** caused slightly decreased anisotropy at lowest concentrations, whereas second enantiomer at highest concentrations.

These results showed that the compounds have practically no influence on fluidity in the hydrophobic region of the lipid bilayers. Slight changes in the values of anisotropy were observed for the highest concentrations of tested compounds relative to the control sample which may be a consequence of modifications observed in the hydrophilic region.

## 3. Materials and Methods 

### 3.1. Chemicals

*N*-bromosuccinimide (NBS, purity ≥ 95%) was purchased from Sigma-Aldrich (St. Louis, MO, USA). Analytical grade acetic acid, sodium hydrogen carbonate, anhydrous magnesium sulfate, sodium chloride (35–37%), tetrahydrofuran (THF), acetone, and hexane were purchased from Chempur (Piekary Śląskie, Poland). Hexane was purified by distillation before use for column chromatography. Silica gel used for column chromatography (Kieselgel 60, 230–400 mesh) was purchased from (Merck, Darmstadt, Germany).

Enantiomers of (*E*)-3-(2′,5′-dimethylphenyl)hex-4-enoic acid (**4**), both with ee > 99%, were obtained previously by our working group in two-step synthesis from the corresponding enantiomeric (*E*)-4-(2′,5′-dimethylphenyl)but-3-en-2-ols [24].

### 3.2. Analysis

Measurements of melting points (uncorrected) were carried out on a Boetius apparatus (Nagema, Dresden, Germany). Specific optical rotations were determined using a P-2000-Na digital polarimeter with intelligent Remote Module (iRM) controller (Jasco, Easton, MD, USA).

Analytical Thin Layer Chromatography (TLC) was carried out on aluminium plates covered with a silica gel (DC-Alufolien Kieselgel 60 F_254_, Merck, Darmstadt, Germany) using mixture of hexane/acetone (3:1, *v*/*v*) as the developing system. Visualization reagent was a solution of 1% Ce(SO_4_)_2_ and 2% H_3_[P(Mo_3_O_10_)_4_] in 10% H_2_SO_4_.

Gas chromatography (GC) was performed on Agilent Technologies 6890N instrument (Santa Clara, CA, USA) equipped with autosampler and FID detector using hydrogen as a carrier gas. Progress of bromolactonization and the purity of isolated compounds was checked on DB-5HT column (Agilent, Santa Clara, USA, polyimide-coated fused silica tubing, 30 m × 0.25 mm × 0.10 × μm) under the following conditions, injector 200 °C, detector 280 °C, initial column temperature: 100 °C, 100–200 °C (20 °C min^−1^), 200–300 °C (30 °C min^−1^), final column temperature 300 °C (1 min). Total time of analysis 9.3 min.

Chiral Gas Chromatography (CGC) was carried out on CP Chirasil-DEX CB column (Agilent, Santa Clara, CA, USA, cyclodextrin bonded to dimethylpolysiloxane, 25 m × 0.25 mm × 0.25 μm) with a temperature program as follows, injector 280 °C, detector 250 °C; initial column temperature 50 °C, 50–200 °C (0.5 °C min^−1^), final column temperature 200 °C (1 min). Total time of analysis 301 min.

Nuclear magnetic resonance spectra (^1^H NMR, ^13^C NMR, HMQC, and HMBC) were recorded on a Bruker Avance II 600 MHz spectrometer (Bruker, Rheinstetten, Germany) for CDCl_3_ solutions, with signals of residual solvent (δ_H_ = 7.26, δ_C_ = 77.0) as references for chemical shifts. Infrared spectroscopy (IR) was conducted using Nicolet iS10 FTIR Spectrometer (Thermo Scientific™, Waltham, MA, USA) equipped with monolithic diamond ATR crystal attachment. High-resolution mass spectra (HRMS) were recorded using electron spray ionization (ESI) technique on spectrometer Waters ESI-Q-TOF Premier XE (Waters Corp., Millford, MA, USA). 

X-ray data were collected on a Xcalibur Sapphire2 diffractometer (Mo-Kα radiation; λ = 0.71073 Å). X-ray data were collected at 100 K using an Oxford Cryosystem device. Data reduction and analysis were carried out with the CrysAlis ‘RED’ or CrysAlisPro program (Oxford Diffraction, Wrocław (Poland), 2001, 2003). Analytical numeric absorption correction using a multifaceted crystal model based on expressions derived by Clark and Reid [43] was applied. Space groups were determined, based on systematic absences and intensity statistics. The structures were solved by direct methods using the SHELXS program and refined using all *F*^2^ data, as implemented by the SHELXL97 program [44]. Non-hydrogen atoms were refined with anisotropic displacement parameters. All H atoms were placed at calculated positions. Before the last cycle of refinement all H atoms were fixed and were allowed to ride on their parent atoms. The absolute configurations were confirmed by anomalous dispersion effects in diffraction measurements on the crystal. Crystal data for (+)-(4*R*,5*R*,6*S*)-**5** and (−)-(4*S*,5*S*,6*R*)-**5** reported in this paper, have been deposited with the Cambridge Crystallographic Data Centre (CCDC) as supplementary publication numbers 1874584 and 1874585, respectively. Copies of the data can be obtained, free of charge, on application to CCDC, 12 Union Road, Cambridge CB12 1EZ, UK (fax +44-1223-336033 or e-mail: deposit@ccdc.cam.ac.uk).

### 3.3. Synthesis of Bromolactones ***5***–***7***—General Procedure

Acid **4** (6.9 mmol), NBS (14 mmol) and a drop of acetic acid were dissolved in 70 mL of THF and the reaction mixture was stirred at room temperature. When the substrate reacted completely (48h, TLC, GC), the mixture was successively washed with saturated solutions of NaHCO_3_ and NaCl. Organic layer was separated and dried over anhydrous MgSO_4_. After evaporation of solvent in vacuo the products were separated by silica gel column chromatography using hexane followed by hexane/acetone, 20:1, as elution systems.

Bromolactonization of (+)-(*S*,*E*)-3-(2′,5′-dimethylphenyl)hex-4-enoic acid (**4**) (1.51 g, 6.9 mmol) afforded products with physical and spectral data given below:

*(+)-(4R,5R,6S)-5-t-Bromo-4-r-(2′,5′-dimethylphenyl)-6-c-methyltetrahydropyran-2-one* (**5**): Yield 24% (0.486 g), colourless crystals, mp = 113–120 °C, *R*_f_ = 0.33 (hexane/acetone, 3:1), ee > 99%; *t*_R_ = 213.3 min, ${\left[\alpha \right]}_{D}^{20}$ = + 11.5 (c 0.25; CH_2_Cl_2_); IR (solid, cm^−1^): 1720 (s), 1506 (w), 1231 (s), 1037 (s), 972 (m), 820 (m), 632 (m); ^1^H NMR (600 MHz, CDCl_3_), *δ* 1.68 (d, *J* = 6.0 Hz, 3H, CH_3_-7), 2.32, 2.33 (two s, 6H, CH_3_-2′, CH_3_-5′), 2.63 (dd, *J* = 18.0, 10.8 Hz, 1H, one of CH_2_-3), 2.97 (dd, *J* = 18.0, 6.6 Hz, 1H, one of CH_2_-3), 3.74 (td, *J* = 10.8, 6.6 Hz, 1H, H-4), 4.05 (t, *J* = 10.8 Hz, 1H, H-5), 4.68 (dq, *J* = 10.8, 6.0 Hz, 1H, H-6), 6.92 (s, 1H, H-6′), 7.01 (dd, *J* = 7.8, 1.2 Hz, 1H, H-4′), 7.08 (d, *J* = 7.8 Hz, 1H, H-3′); ^13^C NMR (151 MHz, CDCl_3_) *δ* 19.2 (CH_3_-2′), 20.7 (C-7), 21.2 (CH_3_-5′), 37.7 (C-3), 41.8 (C-4), 54.2 (C-5), 80.1 (C-6), 125.6 (C-6′), 128.3 (C-4′), 130.7 (C-3′), 132.6 (C-2′), 136.5 (C-5′), 138.8 (C-1′), 169.2 (C-2); HRMS: calcd for C_14_H_17_BrO_2_ [M + Na]^+^: 319.0309, found 319.0307.

Crystal data for (+)-(4*R*,5*R*,6*S*)-***5***: C_14_H_17_BrO_2_, *M* = 297.18, orthorhombic, *P*2_1_2_1_2_1_, *a* = 6.129(2), *b* = 10.839(3), *c* = 19.596(3) Å, *V* = 1301.8(6) Å^3^, *Z* = 4, *D*_c_ = 1.516 Mg·m^−3^, *T* = 100(2) K, *R* = 0.024, *wR* = 0.044 (3637 reflections with *I* > 2σ(*I*)) for 154 variables, Flack parameter: 0.000(4); CCDC 1874584.

*(−)-cis-(4R,5R,6S)-5-(1-Bromoethyl)-4-(2′,5′-dimethylphenyl)dihydrofuran-2-one* (**6**): Yield 7% (0.135 g); colourless solid; mp = 110–115 °C, *R*_f_ = 0.47 (hexane/acetone, 3:1), ee > 99%, *t*_R_ = 196.6 min; ${\left[\alpha \right]}_{D}^{20}$ = − 63.5 (*c* 0.5, CH_2_Cl_2_); IR (solid, cm^−1^): 1774 (s), 1506 (w), 1185 (s), 1147 (s), 989 (m), 823 (m), 629 (m); ^1^H NMR (600 MHz, CDCl_3_) *δ* 1.80 (d, *J* = 6.6 Hz, 3H, CH_3_-7), 2.29 (s, 3H, CH_3_-5′), 2.37 (s, 3H, CH_3_-2′), 2.61 (dd, *J* = 18.0, 1.8 Hz, 1H, one of CH_2_-3), 3.09 (dd, *J* = 18.0, 9.0 Hz, 1H, one of CH_2_-3), 3.80 (dq, *J* = 9.6, 6.6 Hz, 1H, H-6), 4.11 (ddd, *J* = 9.0, 6.0, 1.8 Hz, 1H, H-4), 4.75 (dd, *J* = 9.6, 6.0 Hz, 1H, H-5), 6.86 (s, 1H, H-6′), 7.00 (d, *J* = 7.8 Hz, 1H, H-4′), 7.06 (d, *J* = 7.8 Hz, 1H, H-3′); ^13^C NMR (150 MHz, CDCl_3_) *δ* 19.9 (CH_3_-2′), 21.2 (CH_3_-5′), 23.4 (C-7), 38.4 (C-3), 38.5 (C-4), 45.3 (C-6), 86.8 (C-5), 126.4 (C-6′), 128.5 (C-4′), 130.9 (C-3′), 133.3 (C-2′), 136.1 (C-5′), 136.4 (C-1′), 176.4 (C-2); HRMS: calcd for C_14_H_17_BrO_2_ [M + Na]^+^: 319.0309, found 319.0305.

*(+)-trans-(4R,5S,6R)-5-(1-Bromoethyl)-4-(2′,5′-dimethylphenyl)dihydrofuran-2-one* (**7**): Yield 11% (0.233 g); dense, colourless liquid; *R*_f_ = 0.38 (hexane/acetone, 3:1), ee = 99%, *t*_R_ = 212.9 min; ${\left[\alpha \right]}_{D}^{20}$ = + 3.8 (*c* 1.0, CH_2_Cl_2_); IR (liquid, cm^−1^): 1779 (s), 1505 (w), 1151 (s), 1006 (s), 811 (m), 634 (m); ^1^H NMR (600 MHz, CDCl_3_) *δ* 1.69 (d, *J* = 7.2 Hz, 3H, CH_3_-7), 2.34 (s, 3H, CH_3_-5′), 2.38 (s, 3H, CH_3_-2′), 2.54 (dd, J = 18.6, 6.6 Hz, 1H, one of CH_2_-3), 3.14 (dd, *J* = 18.6, 10.2 Hz, 1H, one of CH_2_-3), 3.95 (ddd, J = 10.2, 6.6, 5.4 Hz, 1H, H-4), 4.33 (qd, *J* = 7.2, 5.4 Hz, 1H, H-6), 4.67 (t, *J* = 5.4 Hz, 1H, H-5), 7.01–7.03 (m, 2H, H-4′, H-6′), 7.09 (d, *J* = 7.8 Hz, 1H, H-3′); ^13^C NMR (151 MHz, CDCl_3_) *δ* 19.4 (CH_3_-2′), 21.1 (CH_3_-5′), 21.5 (C-7), 37.7 (C-3), 38.9 (C-4), 50.1 (C-6), 88.6 (C-5), 126.3 (C-6′), 128.1 (C-4′), 130.8 (C-3′), 131.9 (C-2′), 136.8 (C-5′), 139.8 (C-1′), 175.2 (C-2); HRMS: calcd for C_14_H_17_BrO_2_ [M + Na]^+^: 319.0309, found 319.0311.

Bromolactonization of (*−*)-(*R*,*E*)-3-(2′,5′-dimethylphenyl)hex-4-enoic acid **4** (1.19 g, 5.47 mmol) afforded products with following data:

*(−)-(4S,5S,6R)-5-t-Bromo-4-r-(2′,5′-dimethylphenyl)-6-c-methyltetrahydropyran-2-one (**5**):* Yield 16% (0.75 g); colourless crystals, mp 121−125 °C; ee > 99%; *t*_R_ = 212.7 min, ${\left[\alpha \right]}_{D}^{20}$ = − 11.5 (*c* 0.5, CH_2_Cl_2_); spectroscopic data identical to those reported herein for (+)-(4*R,*5*R,*6*S)-***5**.

Crystal data for (−)-(4*S*,5*S*,6*R*)-**5**: C_14_H_17_BrO_2_, 297.18, orthorhombic, *P*2_1_2_1_2_1_, *a* = 6.142(2), *b* = 10.872(3), *c* = 19.627(3) Å, *V* = 1310.6(6) Å^3^, *Z* = 4, *D*_c_ = 1.506 Mg·m^−3^, *T* = 100(2) K, *R* = 0.046, *wR* = 0.111 (5312 reflections with *I* > 2σ(*I*)) for 154 variables, Flack parameter: – 0.007(8); CCDC 1874585.

*(+)-cis-(4S,5S,6R)-5-(1-Bromoethyl)-4-(2′,5′-dimethylphenyl)dihydrofuran-2-one* (**6**): Yield 10% (0.156 g); colourless solid, mp 113−119 °C; ee > 99%; *t*_R_ = 194.8 min, ${\left[\alpha \right]}_{D}^{20}$ = + 63.5 (*c* 0.5, CH_2_Cl_2_); spectroscopic data identical to those reported herein for (−)-*(*4*R,*5*R,*6*S)*-**6**.

*(−)-trans-(4S,5R,6S)-5-(1-Bromoethyl)-4-(2′,5′-dimethylphenyl)dihydrofuran-2-one* (**7**): Yield 11% (0.174 g); dense, colourless liquid; ee = 99%; *t*_R_ = 211.1 min, ${\left[\alpha \right]}_{D}^{20}$ = − 3.8 (*c* 1.0, CH_2_Cl_2_); spectroscopic data identical to those reported herein for (+)-(4*R,*5*S,*6*R)-***7**.

### 3.4. Cell Lines

D17 cell line (canine osteosarcoma) was obtained from American Type Culture Collection (ATCC, Rockville, MD, USA). The B cell lymphoma cell line (CLBL-1) was obtained from Barbara C. Rütgen, Institute of Immunology, Department of Pathobiology of the University of Veterinary Medicine, Vienna, Austria [45]. The canine cell line GL-1 (B cell leukemia) was obtained from Yasuhito Fujino and Hajime Tsujimoto from the University of Tokyo, Department of Veterinary Internal Medicine, Tokyo, Japan [46]. CLB70 line (B cell chronic leukemia) was established by one of the coauthors of the manuscript [47]. The human Jurkat cell line (T cell leukemia), coming from ATCC was kindly provided from the collection of the Institute of Immunology and Experimental Therapy, Polish Academy of Sciences, Wroclaw, Poland. The cultures were maintained in CO_2_ incubator in a humidified atmosphere at 37 °C using RPMI 1640 medium (Institute of Immunology and Experimental Therapy, Wroclaw, Poland) supplemented with 2 mM l-glutamine, 100 U/mL penicillin, 100 µg/mL streptomycin, and 10% fetal bovine serum (FBS) (Sigma-Aldrich, Steinheim, Germany) for GL-1, D17 and Jurkat cells and 20% FBS for CLBL-1 and CLB70 cells. To keep at an optimal density (50–70% confluence) cells were cultured in 25 cm^2^ cell flasks (Corning Inc., Corning, NY, USA) and subcultivated every other day. 

### 3.5. Determination of Antiproliferative Activity

Cytotoxicity of the tested bromolactones was determined using MTT assay. Initial solutions of these compounds at a concentration of 10 mg/mL in DMSO (POCH, Gliwice, Poland) were further diluted using the culture media as a solvent to prepare solutions within a concentration range of 0.05 to 50 μg/mL (DMSO concentration was less than 1% in each dilution). The details of the procedure were described in our previous work [24].

### 3.6. Hemolytic Activity

In order to determine the cytotoxicity of tested compounds, a hemolytic activity test was performed using pig red blood cells (RBCs). The method applied was based on that described by Łuczyński et al. [48]. The hemolytic activity of the compounds was determined as ethanolic solutions at concentrations 10, 20, 40, 60, 80, and 100 µM. The control sample contained only ethanol in the same amounts as the samples tested. The final concentration of erythrocytes in the sample was 1.2% hematocrit. Hemolysis caused by the compounds was determined on the basis of the hemoglobin concentration released from red blood cells. The measurement was performed at 540 nm using a UV–Vis spectrophotometer (Specord 40, AnalytikJena, Jena, Germany). 

### 3.7. Fluorescence Spectroscopy

The interaction of tested compounds with red blood cells membranes (RBCM) was investigated based on a fluorimetric method described earlier by Włoch et al. [49]. RBCM were prepared according to the procedure described in the work of Dodge et al. [50]. The impact of compounds on fluidity and packing arrangement of lipids in RBCM were studied. Fluorescence intensity was measured using three fluorescent probes: MC540 (Merocyanine 540), Laurdan (6-dodecanoyl-2-dimethylaminonaphthalene) and DPH (1,6-diphenyl-1,3,5-hexatriene) located in different areas of the membrane. Concentration of fluorescent probes in the samples was 1 µM. The test concentrations of the compounds were 10, 20, 40, 60, 80, and 100 µM. The control sample contained ethanol in same amounts as the samples tested. Measurements was carried out at 37 °C after one hour of incubation, conducted with a fluorimeter (Cary Eclipse, Varian, San Diego, CA, USA) and performed in three independent experiments.

## 4. Conclusions

Bromolactonization of enantiomeric (*E*)-3-(2′,5′-dimethylphenyl)hex-4-enoic acids (**4**) afforded three enantiomeric pairs of β-(2′,5′-dimethylphenyl)-bromolactones: γ-bromo-δ-lactone (**5**), *cis* δ-bromo-γ-lactone (**6**), and *trans* δ-bromo-γ-lactone (**7**), with defined configurations of stereogenic centers. They showed significant cytotoxicity against canine cancer cell lines, the most potent were enantiomers of *trans* γ-lactone **7**. In all cases higher activity was found for enantiomers obtained from (*R*)-acid **4**. On the other hand, all tested bromolactones did not exhibit cytotoxic activity towards erythrocytes. The results of fluorescence spectroscopy suggest that these compounds concentrate mainly in the hydrophilic part of erythrocyte membrane but have practically no influence on fluidity in the hydrophobic region. The differences in interactions with the membrane between particular enantiomers were observed only for γ-lactones; stronger interactions were found for enantiomer 4*R*,5*R*,6*S* of *cis* γ-lactone **6** and for enantiomer 4*S*,5*R*,6*S* of *trans* γ-lactone **7**

Preliminary studies presented in this work indicate that tested bromolactones are potentially good candidates as anticancer drugs and could be used in future in pharmacological preparations. 

## Figures and Tables

**Table 1:** Antiproliferative activity of synthesized bromolactones **5**–**7** and iodolactones **9**,**10** ^1^ against the selected cancer cell lines. ^2^

Entry	Compound	IC_50_ [μg/mL] ^3^				
		D17	CLBL-1	CLB70	GL-1	Jurkat
1	(4*R,*5*R,*6*S*)-**5**	>50	45.86 ± 3.50	31.30 ± 4.20	31.10 ± 3.89	32.51 ± 2.1
2	(4*S,*5*S,*6*R*)-**5**	>50	33.35 ± 0.40	21.39 ± 5.40	15.65 ± 1.20	30.80 ± 4.90
3	(4*R,*5*R,*6*S*)-**6**	>50	33.27 ± 2.40	19.61 ± 3.70	14.74 ± 2.87	22.47 ± 2.30
4	(4*S,*5*S,*6*R*)-**6**	>50	30.98 ± 1.90	13.21 ± 2.30	8.51 ± 0.81	14.48 ± 5.20
5	(4*R,*5*S,*6*R*)-**7**	>50	18.09 ± 3.33	20.08 ± 0.32	14.92 ± 0.48	16.73 ± 3.40
6	(4*S,*5*R,*6*S*)-**7**	34.31 ± 1.88	14.33 ± 2.53	15.80 ± 0.15	6.97 ± 0.45	9.80 ± 2.10
7	(4*R,*5*R,*6*S*)-**9**	19.39 ± 2.60	8.07 ± 1.21	n.i. ^4^	25.65 ± 4.3	29.40 ± 1.66
8	(4*S,*5*S,*6*R*)-**9**	26.57 ± 3.54	8.01 ± 0.96	n.i.	20.48 ± 3.95	33.84 ± 6.85
9	(4*R,*5*S,*6*R*)-**10**	14.81 ± 3.39	7.10 ± 0.65	n.i.	14.24 ± 6.06	14.30 ± 3.72
10	(4*S,*5*R,*6*S*)-**10**	16.99 ± 4.88	4.76 ± 0.52	n.i.	16.30 ± 4.69	16.16 ± 4.73
11	carboplatin	13.10± 4.30	n.i.	n.i.	7.06± 1.15	21.37± 4.00

^1^ Data for iodolactones (entries 7–10) reported in previous paper [24]; ^2^ Results obtained from more than three independent experiments (four wells each) and expressed as the mean value ± SD (standard deviation); ^3^ IC_50_: anticancer drug concentration inhibiting cell viability by 50%; ^4^ n.i.: not investigated.
